# Contemporary Teaching Approches in Pharmacology

**Published:** 2008-10

**487**

**Prototype software to sensitize medical undergraduate students to animal research methodology**

Raveendran R

**JIPMER, Pondicherry, India.**

In India, medical undergraduate students (UGs) are encouraged to do short term research projects. Since the UGs are exposed to research for the very first time, they need to be sensitized, especially when they take up research involving animals. With this in mind, a prototype software has been developed to introduce the UGs to research involving animals and ethics of animal experimentation. Using the software, the UGs can conduct a simulated experiment with a hypothetical new drug to find out its effects on the rabbit eye. They will follow the steps of research such as pilot study, sample size calculation, randomization, blinding, control and filling up forms for necessary permissions. The software will enable them to collect data by doing the experiment on the screen, tabulate and analyse the data statistically and draw conclusions. Further the software includes information about 3R principles, ethical requirements, the governmental guidelines on animal experiments and various aspects of research for self-learning. Using live animals for the sole purpose of teaching research methodology to UGs is not ethically sound, but the software can be used as an alternative. Sensitizing the UGs to the ethical principles of animal experimentation will go a long way in refining the methods and reducing the number of animals when they conduct live animal experiments. Further the knowledge of various concepts of research methodology and statistics imparted by the software will be useful to UGs when they embark on actual research projects later in their career.

**488**

**A comparative study of problem based learning versus lecture based learning in medical students of AIMST University, Malaysia**

Patil S^1^, Ameya H^2^

**^1^GSL Medical college, Rajahmundry, India, ^2^AIMST University, Sungai Petani, Malaysia.**

During the period from May to July 2008, the Problem Based Learning (PBL) coordinators of the School of Medicine, AIMST University conducted a controlled prospective study of PBL versus Lecture Based Learning (LBL) in cardiovascular system. Sixty four students of year 2 term 1, were randomly assigned to PBL group (n = 32) and to the LBL group (n = 32). Analysis included marks secured by the students in the continuous assessment (CA) examination (consisting of multiple-choice questions, true and false questions, short answer and long answer questions) and feedback regarding teaching methodology from the students in both the groups. It was found that the 80% students in the PBL group secured higher marks in the CA examination as compared to LBL group. The feedback results showed that students considered PBL to be an effective and favourable learning method over the LBL. PBL is an effective way of delivering medical education in a coherent, integrated programme and offers several advantages over traditional teaching methods. PBL is a group teaching method that combines the acquisition of knowledge with the development of skills and attitudes.

**489**

**Learning of medical pharmacology via innovation: A student’s perspective**

Srivastava B^1^, Gaur S^1^, Shakur RA^1^, Sharma RN^2^, Sinha Indu^3^

**^1^UHFT Medical College Haldwani, Nainital, ^2^R.D. Gardi Medical College, Ujjain, ^3^PMCH Patna India.**

**Objective:**

To determine the opinion of students regarding the subject pharmacology, teaching methodology, reforms to be introduced, computer and Internet use and its application in teaching learning process.

**Materials and Methods:**

372 students of 2^nd^ professional M.B.BS of UFHT Medical College Haldwani were given a questionnaire which consisted of 2-10 options. They were asked to tick the options and suggestions were also invited. Questionnaire consisted of 3 parts. First part was about demographic characteristic, second part was about the subject and third about the computer or internet use in teaching and learning methods.

**Result:**

372 students in the age group 19-24 yr with male female ration 1:1, mostly from northern India were included in the study. 50.53% (n = 141) considered pharmacology useful and important, 35.48 %(132) wanted more of clinical pharmacology and problem based learning, 21.51 %(80) wanted more frequent use of audiovisual aids. 96.77 %(360) wanted integrated teaching curriculum. 88.17%(328) used computer and 80.64%(300) used internet and 44.64%(192) had knowledge about computer application and telemedicine. 40.86%(152) considered student seminars as useless.

**Conclusion:**

Students appreciated the subject pharmacology and wanted more of integrated problem based learning. So a need was felt for a revised clinical pharmacology oriented curriculum with a computer refresher course. Adoption of such a curriculum will make the subject more relevant and meaningful to the students and medical sciences.

**490**

**Facilitation and evaluation of students learning**

Mittal R, Patil PA, Hiremath SV, Torgal SS, Hogade AP, Bushan A, Hashilkar NK, Majagi SI, Kamat A

**J N Medical College, Belgaum, India.**

**Introduction:**

Various methods of teaching are lectures, tutorials, demonstrations etc but all these involve passive learning, compelling to assess learning outcomes. Increased emphasis is being laid on students centered and integrated curriculum where vertical and horizontal integrated teaching is a relative novel method of training students recommended by the medical council of India. Its superiority over other existing methods in facilitating students learning needs to be assessed and hence the present study was undertaken.

**Methods:**

A 3 year study performed in MBBS Phase -II students of 3 batches. Integrated teaching (seminar on various segments of a topic) was carried out in 3 different ways to each batch of students. Batch 1: Subject experts (faculty) delivered talk on segments of topic allotted.

**Batch 2:**

Randomly selected 7-10 students presented the topic (it was guided by faculty).

**Batch 3:**

similar to batch 2 but here a pre-session test (validated mcq’s) was conducted. About 10 topics were covered in a year (same for all the batches) and were announced 15days prior to the seminar for the student to prepare. The seminar was for 2 hrs and a post -session was conducted using pre- validated mcq.s following it to asses the learning outcome.

**Results:**

Post-sessions scores (mean +SD) of all batches was calculated and was analysed by ANOVA. There was a significant improvement in the performance of batch 3 as compared to other batches.

**Conclusion:**

Pre-session tests promote students participation in teaching learning activities and also facilitates the learning process.

